# GLP-1 Gene-Modified Human Umbilical Cord Mesenchymal Stem Cell Line Improves Blood Glucose Level in Type 2 Diabetic Mice

**DOI:** 10.1155/2019/4961865

**Published:** 2019-12-27

**Authors:** Ying Chang, Mingxin Dong, Yan Wang, Haotian Yu, Chengbiao Sun, Xin Jiang, Wei Chen, Xin Wang, Na Xu, Wensen Liu, Ningyi Jin

**Affiliations:** ^1^Medical College, Yanbian University, Yanji 133002, China; ^2^Institute of Military Veterinary Medicine, Academy of Military Medical Sciences, Key Laboratory of Jilin Province for Zoonosis Prevention and Control, Changchun 130122, China; ^3^Jilin Medical University, Jilin 132013, China; ^4^Jilin Agricultural University, Changchun 130000, China

## Abstract

Type 2 diabetes constitutes a serious threat to the health of patients, but there is currently no ideal treatment in the clinic. Glucagon-like peptide-1 and human umbilical cord mesenchymal stem cells have been confirmed to have antidiabetic effects, but both of them have certain defects in the process of antidiabetes, which cannot meet the need of clinical treatment. We hypothesized that human umbilical cord mesenchymal stem cells can be used as a vector to construct a novel cell line that expresses GLP-1 in vivo for a long time. And this cell strain results in lowering blood glucose in type 2 diabetic mice. The results showed that after 3 weeks of intramuscular injection of the new cell line, the fasting blood glucose of type 2 diabetic mice returned to the normal range, and the hypoglycemic effect was maintained within 3 weeks after putting an end to the drug. At the same time, during the administration, the mice lost weight, the food intake decreased, the half-life of GLP-1 in the body prolonged, the IR reduced, and the pancreatic function recovered. The results of this study indicate that the novel cell line can prolong the half-life of GLP-1 in vivo and effectively lower blood sugar, which is a feasible method to improve type 2 diabetes.

## 1. Introduction

Type 2 diabetes mellitus (T2DM) is a chronic disease marked by hyperglycemia. It is generally thought that its pathogenesis is linked to insulin resistance, insulin secretion defects, obesity, inflammation and other factors [1, 2]. Although many methods have been tested to treat diabetes, including oral antidiabetic drugs, insulin, and stem cells, T2DM is still not cured.

Glucagon-like peptide-1 (GLP-1) is an incretin hormone secreted mainly by intestinal L cells [3, 4]. GLP-1 promotes islet secretion of insulin under the stimulation of glucose, while inhibiting the secretion of glucagon and slowing gastric emptying, reducing appetite, and reducing food intake [5–9]. Therefore, GLP-1 does not lead to hypoglycemia when it plays a hypoglycemic effect. Many scholars believe that GLP-1 is the most promising anti-T2DM drug [10, 11]. However, dipeptidyl peptidase IV (DPP-IV) and rapid renal clearance in the body result in rapid degradation of GLP-1 in plasma, limiting the clinical therapeutic potential of GLP-1 [12, 13].

Human umbilical cord mesenchymal stem cells (hUC-MSCs) are stomatal cells isolated from connective tissue around the umbilical cord blood vessels. It gets the advantages of easy availability, strong proliferative power, and low immunogenicity. It has become one of the clinically available mesenchymal stem cells [14], which can be used not only for clinical treatment but also as a carrier for other drugs [15]. A large number of animal experiments and clinical studies have shown that hUC-MSCs can reduce blood sugar in animals and diabetic patients, improve the pancreatic microenvironment, and promote endogenous islet cell proliferation [16]. However, hUC-MSCs do not improve the body weight and fat ratio in obese diabetic patients.

We used GLP-1 gene modification of human umbilical cord mesenchymal stem cells by adenovirus-infected stem cells and applied it to T2DM mice induced with a high-fat and high-glucose diet combined with streptozotocin to observe its hypoglycemic effect. The mechanism of the hypoglycemic effect is explored. This study is aimed at providing innovative ideas and methods for the treatment of type 2 diabetes.

## 2. Materials and Method

### 2.1. Adenovirus and Human Umbilical Cord Mesenchymal Stem Cell Source

Both adenovirus and human umbilical cord mesenchymal stem cells were donated by Jilin University, in which GLP-1 and GFP genes were coexpressed in adenovirus Ad-GLP-1. Only the GFP gene was written in Ad-GFP.

### 2.2. Construction of Novel Cell Lines

hUC-MSCs were placed in 2 T75cm bottles at 5 × 10^6^ cells/mL, and the required virus volume was calculated by MOI = 30. Ad-GLP-1 and Ad-GFP were added to T75cm bottles according to the calculated virus volume and replaced after 3 hours. After the new culture solution was inoculated for 48 hours, the culture solution was discarded; about 1 mL of trypsin containing no EDTA was added and cultured in a 37°C incubator for 1-2 min, centrifuged, and washed twice with physiological saline; and the number of cells was counted. Two cell lines of Ad-GLP-1-hUC-MSCs and Ad-GFP-hUC-MSCs were prepared.

### 2.3. Animal Experiment

Male 8-week-old C57BL/6J mice (purchased from Liaoning Changsheng) are weighing 20 ± 22 grams. They are housed in a standard animal room (temperature 21-23°C, humidity 50%, and light 12 : 12 h light/dark cycle), with free diet water. The mice were acclimated to the environment for one week before beginning the experiment.

### 2.4. Induced Type 2 Diabetes Model

After giving a four-week high-fat diet (Beijing Huafukang Animal Company), a freshly prepared streptozotocin solution (130 mg/kg) was injected intraperitoneally, and the fasting blood glucose level was measured after one week. Mice with polyuria, polyphagia, and fasting blood glucose values ≥ 7.8 mmol/mL were considered to be successful in the induction of type 2 diabetes.

### 2.5. Treatment Programs

In the experiment, mice were randomly separated into 3 groups according to the following treatment protocol (*n* = 10).

- Group I (*n* = 10): Ad-GFP-hUC-MSC group
- Group II (*n* = 10): Ad-GLP-1-hUC-MSC group
- Group III (*n* = 10) model group

Groups I and II were intramuscularly injected with 5 × 10^5^ Ad-GFP-hUC-MSCs and Ad-GLP-1-hUC-MSCs, respectively, and group III was injected with the same dose of normal saline. Each group of mice was dosed once a week for 3 weeks. After intramuscular injection of Ad-GFP-hUC-MSCs and Ad-GLP-1-hUC-MSCs, blood was collected from the posterior venous plexus at different time points to obtain blood glucose, GLP-1, and insulin values. At the end of the experiment, the collected blood was centrifuged at 3000 rpm for 10 minutes, and serum was taken for correlation detection. The pancreas was collected from each group of mice for morphological examination. All laboratory and animal care procedures are performed in accordance with the regulations approved by the Animal Care and Use Committee of Jilin Medical College (2018-LW010).

### 2.6. Plasma Analysis: Measurement of GLP-1, Insulin, and Blood Glucose

Mice were not fed for 12 hours and blood samples were harvested. Secreted plasma GLP-1 levels and insulin levels were determined by the enzyme-linked immunosorbent assay (ELISA); blood glucose levels in serum were determined by the glucose oxidase assay.

### 2.7. Assessment of Insulin Sensitivity

The extent of insulin resistance was estimated by a steady-state model evaluation of the HOMA-IR score according to the method described by Matthews et al. The HOMA-IR score was calculated using the following formula: fasting plasma glucose (mmol/L) × fasting serum insulin (*μ*U/mL)/22.5 [19].

### 2.8. Glucose Tolerance Test (OGTT)

The mice were fasted for 12 hours, and D-glucose (2 g/kg body weight) was intragastrically administered. The glucose oxidase method was utilized to detect blood glucose levels at 0, 30, 60, 90, and 120 minutes after gavage.

### 2.9. Histological Examination

Mice were intraperitoneally injected with 100 mg/kg bromodeoxyuridine (BrdU; Sigma) 5 hours before killing. The pancreas was removed and fixed in 4% paraformaldehyde for 24 hours and then embedded in paraffin. Sections (3 mm) were obtained and stained with hematoxylin and eosin. Sections were microscopically examined using Nikon (Nikon Eclipse 80i; Tokyo, Japan), and the data was collected by scanning the sections in a random system using a computer with imaging software (ACT-2U version 1.52; Nikon). The islet area and the number of islets were measured blindly.

### 2.10. Statistical Analysis

Data are expressed as mean ± SD and analyzed using SPSS for Windows version 13.0 (SPSS, Chicago, IL, USA). Statistical significance was determined by Student's *t*-test or one-way ANOVA followed by the Student-Newman-Keuls post hoc test. Differences at *p* < 0.05 were considered significant.

## 3. Result

### 3.1. Adenovirus Infection of Mesenchymal Stem Cells

**Figure 1:**
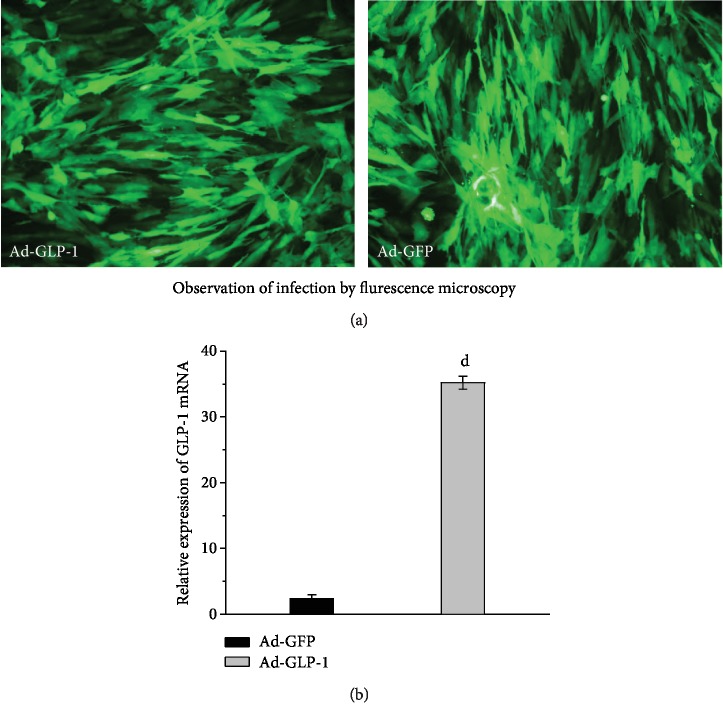
Adenovirus infection of mesenchymal stem cells. (a) Observation of infection by fluorescence microscopy. (b) Real-time PCR was used to detect the relative expression of GLP-1 in cells after adenovirus Ad-GFP and Ad-GLP-1 infection of mesenchymal stem cells for 48 hours. ^d^*p* < 0.01, compared with Ad-GFP-hUC-MSC control mice.

In order to determine whether adenovirus is infected into mesenchymal stem cells, we first observed the mesenchymal stem cells infected with adenovirus for 48 hours by fluorescence microscopy. The results showed that adenoviruses Ad-GFP and Ad-GLP-1 were infected into mesenchymal stem cells. However, there were no significant differences in the degree of infection (Figure 1(a)). The relative expression level of GLP-1 in the cells after 48 hours of virus infection was assessed by real-time PCR. The relative expression of GLP-1 in Ad-GLP-1-infected mesenchymal stem cells was significantly higher than that in Ad-GFP-infected mesenchymal stem cells (Figure 1(b)).

### 3.2. Release of GLP-1 by Modified Mesenchymal Stem Cells in Type 2 Diabetic Mice

**Figure 2:**
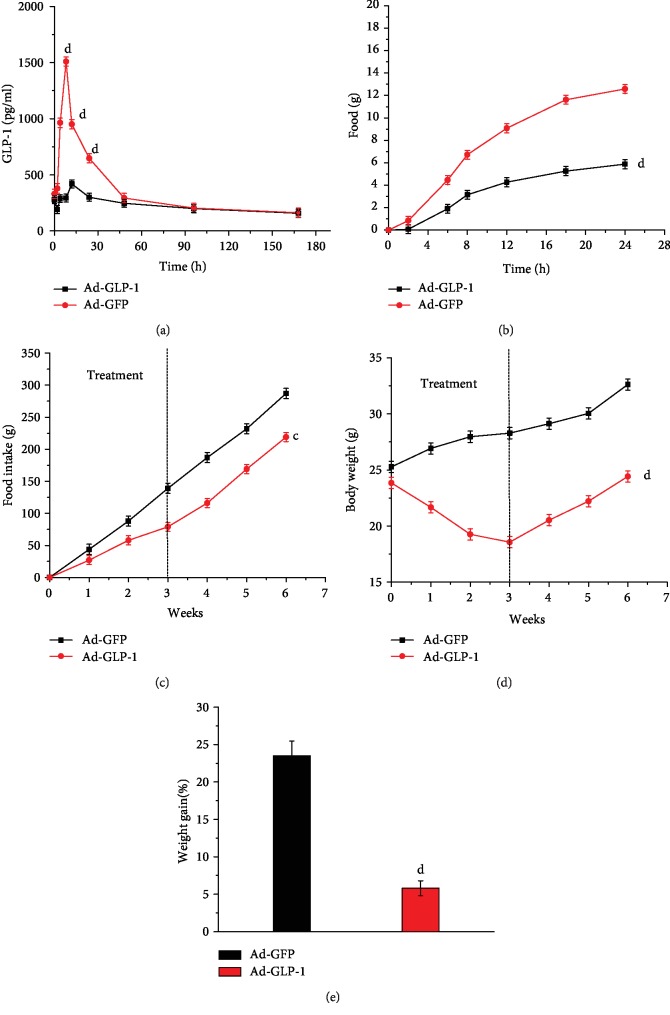
Release of GLP-1 by modified mesenchymal stems cells in type 2 diabetic mice. (a) GLP-1 content in mouse serum within 168 hours after single intramuscular injection of modified mesenchymal stem cells. (b) Cumulative food intake 24 hours after a single intramuscular injection. (c) Effects on food intake at different times after intramuscular injection. (d) The effect of different times on body weight. (e) Weight gain ratio at 3 weeks of administration. Data are mean ± SD (*n* = 10). ^c^*p* < 0.05, ^d^*p* < 0.01, compared with Ad-GFP-hUC-MSC control mice.

To assess whether adenovirus-infected mesenchymal stem cells release GLP-1 in vivo, we measured GLP-1 levels in serum within 24 hours after intramuscular injection. Two hours after intramuscular injection of Ad-GLP-1-hUC-MSCs and Ad-GFP-hUC-MSCs, the GLP-1 content in the serum of Ad-GLP-1-hUC-MSC mice began to increase and peaked at 8 hours, indicating that Ad-GLP-1-hUC-MSCs can be efficiently secreted in type 2 diabetic mice. The serum GLP-1 content of mice injected with Ad-GFP-hUC-MSCs showed little change. This indicates that adenovirus-infected Ad-GFP-hUC-MSCs are unable to release GLP-1 in mice (Figure 2(a)).

GLP-1 is known to have an effect of inhibiting food intake and reducing body weight. To further evaluate the release of GLP-1 by Ad-GLP-1-hUC-MSCs in mice, we recorded changes in food intake within 24 hours after intramuscular administration and weekly total food intake after dosing and recorded changes in body weight per week. As expected, the food intake and body weight of the mice in the Ad-GLP-1-hUC-MSC group were significantly lower than those in the Ad-GFP-hUC-MSC group. This result again demonstrates that Ad-GLP-1-hUC-MSC can release GLP-1 in mice after intramuscular injection (Figures 2(b), 2(c), 2(d) and 2(e)).

### 3.3. Modified Mesenchymal Stem Cells Improve Fasting Blood Glucose and Glucose Tolerance in Type 2 Diabetic Mice

**Figure 3:**
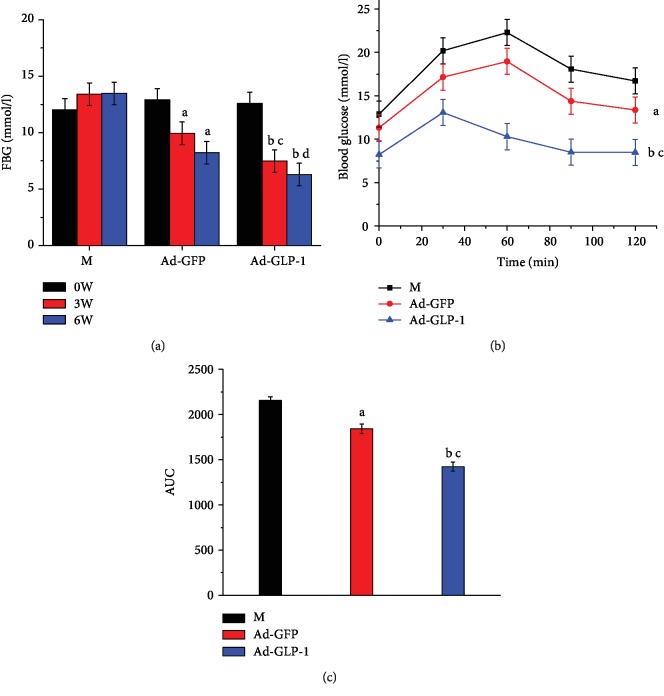
Evaluation of therapeutic effect after cell line injection. Fasting blood glucose levels were measured at 12 hours of fasting, and glucose (2 g/kg) was orally administered to mice. (a) Fasting 12-hour blood glucose of intramuscular injection of Ad-GLP-1 and Ad-GFP at 3 weeks of administration and three weeks of drug withdrawal. (b) Glucose tolerance changes at 3 weeks of administration of Ad-GFP-hUC-MSCs and Ad-GLP-1-hUC-MSCs. (c) Administration of Ad-GFP-hUC-MSCs and Ad-GLP- 1-hUC-MSCs of glucosetolerance at 3 weeks. Data are mean ± SD (*n* = 10). ^a^*p*<0.05, ^b^*p* < 0.01, compared with the diabetes control mice; ^c^*p* < 0.05, ^d^*p* < 0.01, compared with Ad-GFP-hUC-MSC control mice.

To assess the effect of modified mesenchymal stem cells on blood glucose in T2DM mice, we performed a fasting 12-hour blood glucose and glucose tolerance test at 0 week, 3 weeks, and 3 weeks after discontinuation. After 3 weeks of administration, the Ad-GLP-1-hUC-MSC group and the Ad-GFP-hUC-MSC group were able to improve blood glucose and glucose tolerance in type 2 diabetic mice compared with the model group. However, the improvement effect of the Ad-GLP-1-hUC-MSC group was more significant, and the fasting blood glucose level of Ad-GLP-1-hUC-MSCs remained at a lower blood glucose level after 3 weeks of withdrawal. These results indicate that the Ad-GLP-1-hUC-MSC group is more effective in improving blood glucose in type 2 diabetic mice and can be retained for a longer period of time (Figure 3).

### 3.4. Modified Mesenchymal Stem Cells Improve Insulin Sensitivity in Type 2 Diabetic Mice

**Figure 4:**
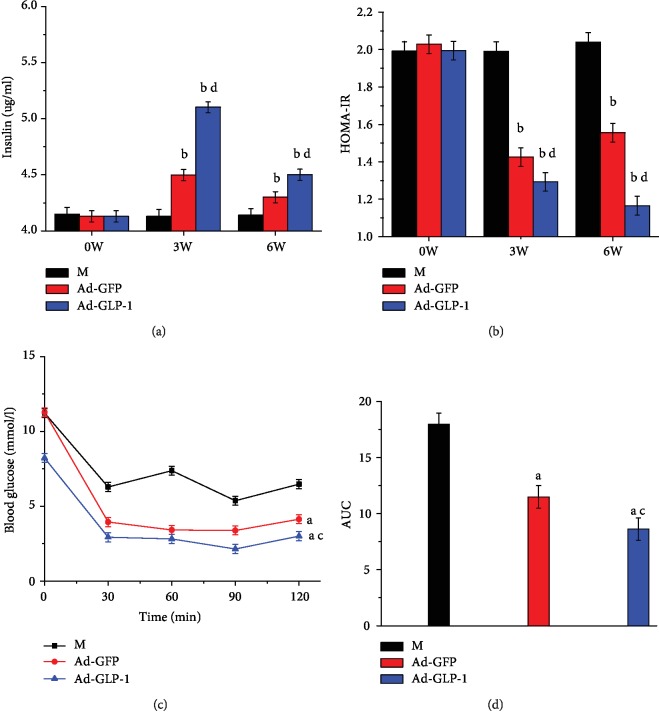
Evaluation of changes in insulin sensitivity after 3 weeks of injection of cell lines. (a) Serum fasting insulin levels were varied in each dose group at 3 weeks of dosing and 3 weeks of drug withdrawal. (b) HOMA-IR changes in each dose group at 3 weeks of administration and 3 weeks after drug withdrawal. (c) Intravenous insulin (0.75 U/kg) was administered to each dose group at 3 weeks of administration, and blood glucose levels were monitored every 30 minutes for 2 hours. (d) Insulin resistance AUC. Data are mean ± SD (*n* = 10). ^a^*p*<0.05, ^b^*p* < 0.01, compared with the diabetes control mice; ^c^*p* < 0.05, ^d^*p* < 0.01, compared with Ad-GFP-hUC-MSC control mice.

To test whether modified mesenchymal stem cells can improve insulin sensitivity in type 2 diabetic mice, we measured insulin and blood glucose levels in fasting mouse serum and scored them according to HOMA-IR. Insulin levels in the Ad-GLP-1-hUC-MSC group were significantly higher than those in the Ad-GFP-hUC-MSC group, but the fasting blood glucose levels were significantly lower. This experiment utilized the HOMA-IR score as an indicator of insulin sensitivity. The mice treated with modified mesenchymal stem cells for 3 weeks had lower HOMA-IR values than the diabetes group (Figures 4(a) and 4(b)). This indicates that the Ad-GLP-1-hUC-MSC group has an effect of improving insulin resistance in type 2 diabetic mice. At the same time, the insulin tolerance test also showed that using insulin is more effective for the Ad-GLP-1-hUC-MSC group than for the Ad-GFP-hUC-MSC group. These results indicate that the Ad-GLP-1-hUC-MSC group improves insulin sensitivity in B cells in type 2 diabetic mice (Figures 4(c) and 4(d)).

### 3.5. Modified Mesenchymal Stem Cells Improve Pancreatic Function in Type 2 Diabetic Mice

**Figure 5:**
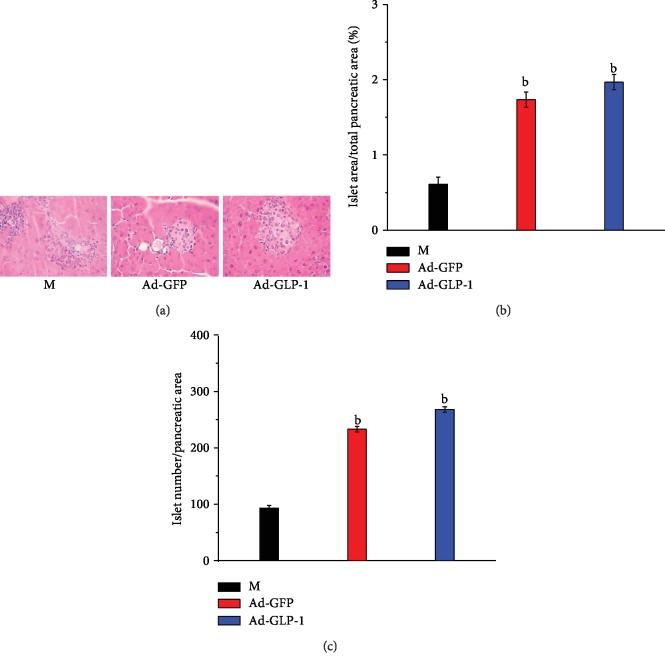
Histological analysis of islets of T2DM mice at 3 weeks of dosing with 2 cell lines. (a) HE staining. Scale: 400. (b) Percent total islet area/total pancreatic area. (c) Total islet number/pancreatic area. ^b^*p* < 0.01, compared with the diabetes control mice.

To determine whether the effect of modified mesenchymal stem cells on fasting blood glucose was associated with pancreatic function recovery, we performed a morphological analysis of the pancreas. After 3 weeks of injection of Ad-GLP-1-hUC-MSCs and Ad-GFP-hUC-MSCs, the morphology of the pancreas in each group was significantly improved compared with that in the model group, and the percent total islet area/total pancreatic area and total islet number/pancreatic area were significantly increased. However, the Ad-GLP-1-hUC-MSC group and the Ad-GFP-hUC-MSC group were basically the same (Figure 5). This suggests that the improvement in fasting blood glucose in T2DM mice may be related to the recovery of islet function.

## 4. Conclusions

The use of plasmids or viruses for gene therapy to maintain GLP-1 levels in plasma is becoming more sophisticated. Intramuscular injection of the adenovirus or plasmid linked to the GLP-1 gene into type 1 diabetic mice reduced the fasting blood glucose level of db/db mice and induced an increase in the number of B cells in mouse islets. Stem cells are regarded as excellent drug carriers because of their low immunogenicity and strong proliferative capacity. In this experiment, we established a highly efficient novel gene delivery system using the characteristics of human umbilical cord mesenchymal stem cells. It produces therapeutic levels of GLP-1 in a mouse model of type 2 diabetes and prolongs the duration of GLP-1 action in vivo.

We constructed new cell lines Ad-GLP-1-hUC-MSCs and Ad-GFP-hUC-MSCs, GLP-1 is highly expressed in the Ad-GLP-1-hUC-MSC cell line, and GLP-1 in type 2 diabetic mice can be released continuously. We examined the therapeutic effects of two cell lines in type 2 diabetic mice. After preparing a mouse model of type 2 diabetes, the new cell line was intramuscularly injected once a week for 3 consecutive times to test the fasting blood glucose of the mice. Fasting blood glucose levels in type 2 diabetic mice injected with the Ad-GLP-1-hUC-MSC cell line returned to normal levels, and after the cell line is stopped, the treatment effect can be maintained for 3 weeks. GLP-1 is glucose dependent, and no hypoglycemic events were observed during drug administration. In the 3-week glucose tolerance test, the area under the curve of the Ad-GLP-1-hUC-MSC group at different time points was smaller than that of the Ad-GFP-1-hUC-MSC group and the model group. This conclusion indicates that the Ad-GLP-1-hUC-MSC group improved the blood glucose of type 2 diabetic mice better than the Ad-GFP-1-hUC-MSC group. This phenomenon may be related to the improvement of pancreatic function and insulin sensitivity. Pancreatic function testing and HOMA-IR support this conclusion.

Previous reports have shown that the long-term effects of GLP-1 on B cell tumor formation and differentiation allow GLP-1 to maintain blood glucose for 16 days in 12-week-old ZDF mice. In our study, although the plasma GLP-1 level decreased rapidly, the improved glucose tolerance of the Ad-GLP-1-hUC-MSC group remained above 21 days, but the number of B cells increased and the B cell function improved. The increase in total islet area/total islet area and total islet/islet area confirms this conclusion.

GLP-1 or its analogs have been demonstrated to improve insulin sensitivity in animal models and human type 2 diabetic patients. The mechanism by which GLP-1 improves insulin sensitivity may involve insulin signaling mediators such as insulin receptor substrate recovery (IRS-1), protein kinase C, Akt activation in muscle or liver, and reduction in hepatic gluconeogenesis. We analyzed the HOMA-IR score, and the improvement of insulin resistance in type 2 diabetic mice after treatment with Ad-GLP-1-hUC-MSCs may be related to the rapid decline in blood glucose levels and increased insulin secretion in mice.

The effects of GLP-1 on food intake include delayed gastric emptying, inhibition of gastric acid secretion, and decreased appetite [13]. In some reports, GLP-1 gene therapy resulted in reduced food intake, decreased weight gain, and decreased glucagon, which is consistent with our experimental results ([Supplementary-material supplementary-material-1]). This effect is mainly in the first few days after administration. The anorexia effect of GLP-1 is thought to occur in the central nervous system and often produces adverse reactions of nausea and vomiting. However, this unfavorable reaction was not found in this mouse experiment.

GLP-1 plays a major role in insulin secretion and synthesis as well as in B cell proliferation and prevention of apoptosis. It has been reported that GLP-1 can stabilize insulin mRNA, increase insulin transcription, and increase the number of islets [17, 18], which is consistent with our experiment result. That is, serum insulin levels after Ad-GLP-1-hUC-MSC cell line treatment are elevated in our experiment. At the same time, several reports have shown that when GLP-1 improves insulin sensitivity, serum insulin levels in treated animals are decreased. These comparative observations may be due to different characteristics of the animal model of diabetes, such as the severity of diabetes and the retention of beta cell function. In this study, we evaluated the efficacy of GLP-1 in the T2DM animal model using an adenovirus vector system. After intramuscular injection of the Ad-GLP-1-hUC-MSC cell line, it can exert the effects of GLP-1 and human umbilical cord mesenchymal stem cells. The fasting blood glucose of type 2 diabetic mice is significantly reduced, the food intake is reduced, and the body weight is improved. In addition, Ad-GLP-hUC-MSC cell lines may improve insulin sensitivity by increasing blood insulin levels and improving fasting blood glucose.

## Data Availability

The data used to support the findings of this study are included within the article.

## Supplementary Materials

Supplementary Materials Supplement 1: glucagon content in the blood of each group of mice at 3 weeks. ^a^*p* < 0.05, ^b^*p* < 0.01, compared with the diabetes control mice; ^c^*p* < 0.05, ^d^*p* < 0.01, compared with Ad-GFP control mice.
