# Supplementary material for: GLP-1 Gene-Modified Human Umbilical Cord Mesenchymal Stem Cell Line Improves Blood Glucose Level in Type 2 Diabetic Mice
Source: Stem Cells Int. 2019 Dec 27;2019:4961865. doi: 10.1155/2019/4961865 (PMC6948274; doi:10.1155/2019/4961865)
Supplement: Supplementary Materials — Supplement 1: glucagon content in the blood of each group of mice at 3 weeks. ap < 0.05, bp < 0.01, compared with the diabetes control mice; cp < 0.05, dp < 0.01, compared with Ad-GFP control mice. [file 4961865.f1.docx]

**Supplement 1**

To evaluate whether the novel cell line has an effect on the glucagon content of type 2 diabetic mice, we measured the glucagon content of each group of mice after 3 weeks of administration, Ad-GLP-1-hUC- MSCs have a better inhibitory effect on glucagon, and this result also indicates that Ad-GLP-1-hUC-MSCs can secrete GLP-1 in vivo.

**

**

Supplement 1. Glucagon content in the blood of each group of mice at 3 weeks . ^a^p< 0.05,^b^p < 0.01, compared with the diabetes control mice;^c^p< 0.05,^d^p < 0.01, compared with Ad-GFP control mice.
